# Defining a Thermodynamic Temperature Scale Using Quantum Dot Heat Engines

**DOI:** 10.3390/e28091016

**Published:** 2026-09-11

**Authors:** Shahaab Mansoor Qureshi, Jan Andries Mol

**Affiliations:** School of Physical and Chemical Science, Queen Mary Universtiy of London, London E1 4NS, UK

**Keywords:** single-electron transistor, quantum dot, heat engine

## Abstract

The ability to define a temperature scale that is independent of any particular material is important for testing quantum thermodynamic concepts at the nanoscale. Here, we present a method for constructing a thermodynamic temperature scale from the reversible operation of quantum dot heat engines. In the limit of vanishing dissipation and lifetime broadening, temperature ratios can be determined directly from measurable open-circuit voltages and combined into a self-consistent temperature scale.

## 1. Introduction

Primary thermometers are sensors that measure temperature by linking it to an observable quantity through a well-known physical law. Examples of primary thermometers include Johnson–Nyquist noise [1] and shot-noise thermometers [2,3,4], Coulomb blockade thermometers, and thermometers based on single-electron devices [5,6,7,8,9,10,11,12,13]. All these thermometers measure the temperature of electrons in a single reservoir without the need for calibration. While primary thermometers determine the temperature of a single reservoir, thermodynamic temperature scales are fundamentally based on temperature ratios. Historically these ratios are established through reversible heat engines and Carnot’s theorem [14]. The question addressed here is whether an analogous construction can be realized using nanoscale quantum heat engines.

The concept of temperature at the nanoscale is itself subtle. Different approaches define local temperature through probe measurements, spectroscopic observables, transport properties, or self-consistent thermodynamic constructions [15,16]. In this work we do not attempt to define a unique local temperature. Instead, we focus on constructing an operational thermodynamic temperature scale from reversible quantum dot heat engines. Recent advances in nanoscale device engineering have enabled the fabrication of quantum dot heat engines that have been used to investigate the equilibrium thermodynamic properties of few-electron quantum systems [17,18], and to probe the efficiency limits of thermal energy harvesters [19,20]. In these nanoelectronic devices, a temperature difference between two reservoirs drives the sequential electron tunneling through a quantum dot in the same way a bias voltage drives sequential electron tunneling in a quantum dot single-electron transistor.

The thermometric use of nanoscale energy-selective conductors is well established, ranging from reversible thermoelectric nanomaterials operating near the Carnot limit and quantum-dot thermometry to quantum thermal machines designed explicitly for temperature measurement. Reversible thermoelectric transport has been analyzed theoretically [21] and quantum thermal machines have been proposed as primary thermometers [22,23,24], quantum dots have been employed as nanoscale thermometers [25,26], and the broader context of thermoelectric transport, quantum heat engines, and quantum thermometry has been reviewed extensively [27,28,29,30,31]. The novelty of the present work is not the thermometric use of a quantum dot itself, nor the observation that energy-selective transport can approach Carnot efficiency. Rather, we demonstrate how a network of quantum dot heat engines may be used to establish self-consistent temperature ratios across multiple reservoirs.

## 2. Results

### 2.1. An Ideal Quantum Dot Heat Engine

**Figure 1:**
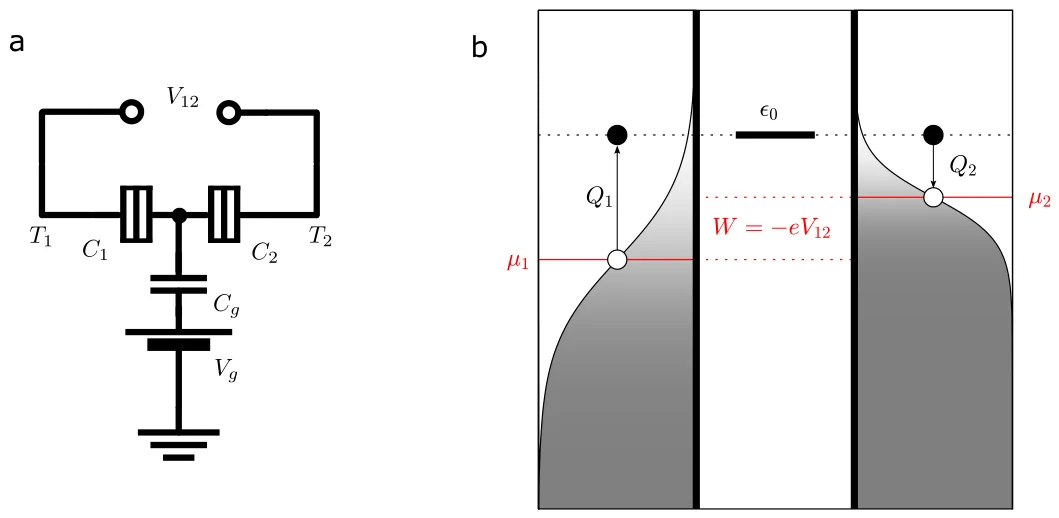
(**a**) Equivalent circuit diagram of a quantum dot heat engine. (**b**) A quantum dot with a single-particle energy level $\epsilon_{0}$ is coupled to two reservoirs (1 and 2) with Fermi distributions set by their respective temperatures. In the open-circuit configuration no current flows and the open-circuit voltage $V_{12}$ ensures that the Fermi distributions at $\epsilon_{0}$ are equal. The work done by the quantum dot heat engine is $W=Q_{1}-Q_{2}$ and is equal to $-eV_{12}$.

We first consider a quantum dot between two reservoirs at temperatures $T_{1}$ and $T_{2}$. The quantum dot heat engine consists of a quantum dot coupled to two reservoirs via tunnel junctions with capacitance $C_{1}$ and $C_{2}$, and an electrostatic gate with capacitance $C_{g}$, as shown in Figure 1a. The electrochemical potential for each of the reservoirs is given by $\mu_{1}=\mu$ and $\mu_{2}=\mu -eV_{12}$, where μ is the electrochemical potential of the reservoirs when they are in thermal equilibrium, *e* the electron charge, and $V_{12}$ the open-circuit voltage between the two reservoirs as illustrated in Figure 1b. The open-circuit voltage is the condition for which the sequential electron tunneling through the quantum dot is zero. In the sequential tunneling limit, the open-circuit condition corresponds to equal forward and reverse tunneling. For a non-interacting single transport level (we assume a large Zeeman splitting or equivalent mechanism that removes spin degeneracy), this is equivalent to requiring equal Fermi occupation probabilities of the two reservoirs at the transport energy $\epsilon_{0}$ [30,32]

(1) $$\frac{1}{e^{\left(\epsilon_{0}-\mu_{1}\right)/k_{B}T_{1}}+1}=\frac{1}{e^{\left(\epsilon_{0}-\mu_{2}\right)/k_{B}T_{2}}+1}$$

which gives

(2) $$-eV_{12}=\left(1-\frac{T_{2}}{T_{1}}\right)\left(\epsilon_{0}-\mu \right).$$

Since the detuning of the single-electron energy level ϵ with respect to the electrochemical potential μ is controlled by voltage applied to the gate, $\epsilon_{0}-\mu =-e\alpha \left(V_{g}-V_{g0}\right)$, where $\alpha =C_{g}/C$ is the gate coupling (with *C* the sum of the gate capacitance $C_{G}$, the source and drain capacitances, $C_{1}$ and $C_{2}$, and any additional capacitances) and $V_{g0}$ is the gate voltage at which the transport level is aligned with the reservoir electrochemical potential in the absence of thermal and electrical bias, the ratio of temperatures can be directly measured from the differential change of the open-circuit voltage as a function of gate voltage

(3) $$\frac{V_{12}}{\alpha (V_{g}-V_{g0})}=1-\frac{T_{2}}{T_{1}}.$$

The offset voltage $V_{g0}$ is obtained from the gate voltage for which the open-circuit voltage vanishes. The gate lever arm α can be determined independently from conventional electrical characterization, for example through Coulomb diamond measurements or finite-bias spectroscopy [33]. Following an established protocol [34], the slopes of the Coulomb diamond are given by $\beta_{1}=-eC_{G}/\left(C-C_{1}\right)$ and $\beta_{2}=eC_{G}/C_{1}$ so that $\alpha =\beta_{1}\beta_{2}/\left(\beta_{1}-\beta_{2}\right)$. Consequently neither parameter requires calibration against a known temperature difference.

**Figure 2:**
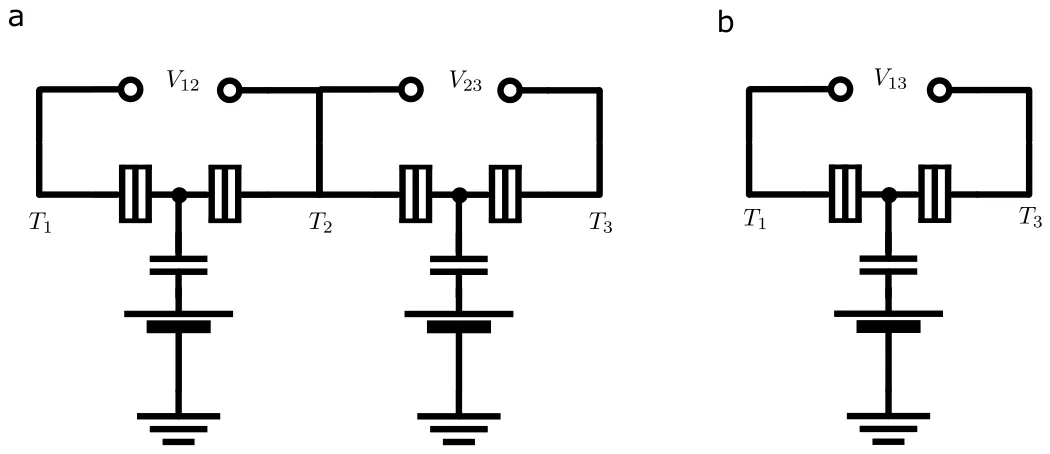
(**a**) Two quantum dot heat engines, one operating between reservoirs at temperatures $T_{1}$ and $T_{2}$, a one between temperatures $T_{2}$ and $T_{3}$. (**b**) A single quantum dot heat engine operating between reservoirs at temperatures $T_{1}$ and $T_{3}$. By combining multiple quantum dot heat engines, a self-consistent temperature scale is determined.

Next, we consider three quantum dot heat engines, *A*, *B*, and *C*, operating between three temperature reservoirs with temperatures $T_{1}$, $T_{2}$, and $T_{3}$, as illustrated in Figure 2. The electrochemical potentials of the three reservoirs are $\mu_{1}$, $\mu_{2}$, and $\mu_{3}$, respectively, and the energy level of each of the quantum dots is $\epsilon_{A}$, $\epsilon_{B}$ and $\epsilon_{C}$. The conditions for zero current are

(4) $$\begin{array}{ccc} \frac{\epsilon_{A}-\mu_{1}}{\epsilon_{A}-\mu_{2}} & = & \frac{T_{1}}{T_{2}}, \end{array}$$

(5) $$\begin{array}{ccc} \frac{\epsilon_{B}-\mu_{2}}{\epsilon_{B}-\mu_{3}} & = & \frac{T_{2}}{T_{3}}, \end{array}$$

(6) $$\begin{array}{ccc} \frac{\epsilon_{C}-\mu_{1}}{\epsilon_{C}-\mu_{3}} & = & \frac{T_{1}}{T_{3}}. \end{array}$$

Multiplying Equation (4) by Equation (5) gives

(7) $$\frac{\epsilon_{A}-\mu_{1}}{\epsilon_{A}-\mu_{2}}\frac{\epsilon_{B}-\mu_{2}}{\epsilon_{B}-\mu_{3}}=\frac{\epsilon_{C}-\mu_{1}}{\epsilon_{C}-\mu_{3}},$$

which is satisfied whenever the three devices are tuned to operate at the same transport energy $\epsilon_{0}$ relative to the relevant electrochemical potential. This does not require physically identical quantum dots but only that their effective transport energies are adjusted to a common value through the gate voltage. Experimentally this condition can be established independently for each device through electrical characterization. Since $\epsilon_{A}-\mu_{2}$ corresponds to the heat absorbed by the second reservoir, and $\epsilon_{B}-\mu_{2}$ the heat rejected by the same reservoir, this means that $T_{2}$ remains unchanged. By tuning the three quantum dot heat engines in this way we find the open-circuit voltage across quantum dot engines *A* and *B*

(8) $$\begin{array}{ccc} -eV_{12} & = & \left(1-\frac{T_{2}}{T_{1}}\right)\left(\epsilon_{0}-\mu +eV_{23}\right), \end{array}$$

(9) $$\begin{array}{ccc} -eV_{23} & = & \left(1-\frac{T_{3}}{T_{2}}\right)\left(\epsilon_{0}-\mu \right), \end{array}$$

Combined they give the open-circuit voltage across quantum dot engine *C*

(10) $$-e\left(V_{12}+V_{23}\right)=\left(1-\frac{T_{3}}{T_{1}}\right)\left(\epsilon_{0}-\mu \right)$$

which does not involve the intermediate temperature $T_{2}$. It follows that, by taking a whole series of quantum dot engines, any range of temperatures may be defined in a self-consistent way that is equivalent to the transitivity of temperature ratios across multiple reservoirs.

### 2.2. A Real Quantum Dot Heat Engine

In contrast to the idealised model discussed in Section 2.1, real quantum dot heat engines operate under conditions where dissipation and finite tunneling rates play a crucial role. To capture these phenomena, we adopt the single-electron transport framework for thermoelectricity, extended to include lifetime broadening and electron–vibrational interactions. This approach accounts for the coupling to a vibrational environment through the reorganization energy λ and incorporates the finite width of the quantum dot energy levels via the lifetime broadening parameter Γ.

The Seebeck coefficient *S* is a key quantity characterizing the thermoelectric response of a real quantum dot heat engine. The thermoelectric response of energy-selective conductors has been extensively studied theoretically and experimentally, including the approach to reversible transport and Carnot efficiency in quantum dot systems [32]. In the linear response regime for vanishing temperature difference, *S* is defined in terms of the open-circuit voltage as

(11) $$S=-\lim_{T_{2}\to T_{1}}\frac{V_{12}}{T_{1}-T_{2}}$$

which in the idealised case described in Section 2.1 would be $S=\left(\epsilon_{0}-\mu \right)/eT_{1}$ but in general can be found by expanding the current in the linear regime as

(12) $$I=GV+G_{\mathrm{th}}\left(T_{1}-T_{2}\right)+\ldots$$

so that the Seebeck coefficient is given by $S=G_{\mathrm{th}}/G$ as

(13) $$S=\frac{1}{T_{1}}\frac{\int \left(\epsilon -\mu \right)f^{\prime }k_{+}d\epsilon \cdot \int \left[1-f\right]k_{-}d\epsilon +\int \left(\epsilon -\mu \right)f^{\prime }k_{-}d\epsilon \cdot \int fk_{+}d\epsilon }{\int f^{\prime }k_{+}d\epsilon \cdot \int \left[1-f\right]k_{-}d\epsilon +\int f^{\prime }k_{-}d\epsilon \cdot \int fk_{+}d\epsilon }$$

where *f* is the Fermi–Dirac distribution. While a general expression for the electron transfer rate, *k*, can be found that combines lifetime broadening and electron–vibrational coupling [35], we will restrict ourselves to the two limiting cases. In the case of lifetime broadening, i.e., when the process is not quasi-static, the electron transfer rates are given by

(14) $$k_{\pm }\propto \frac{1}{{\left(\epsilon -\epsilon_{0}\right)}^{2}+{\left(\Gamma /2\right)}^{2}}.$$

In the case of dissipation as a result of electron–vibrational coupling, the rates are

(15) $$k_{\pm }\propto e^{{\left(\lambda \mp \left(\epsilon -\epsilon_{0}\right)\right)}^{2}/4\lambda k_{B}T_{1}}.$$

**Figure 3:**
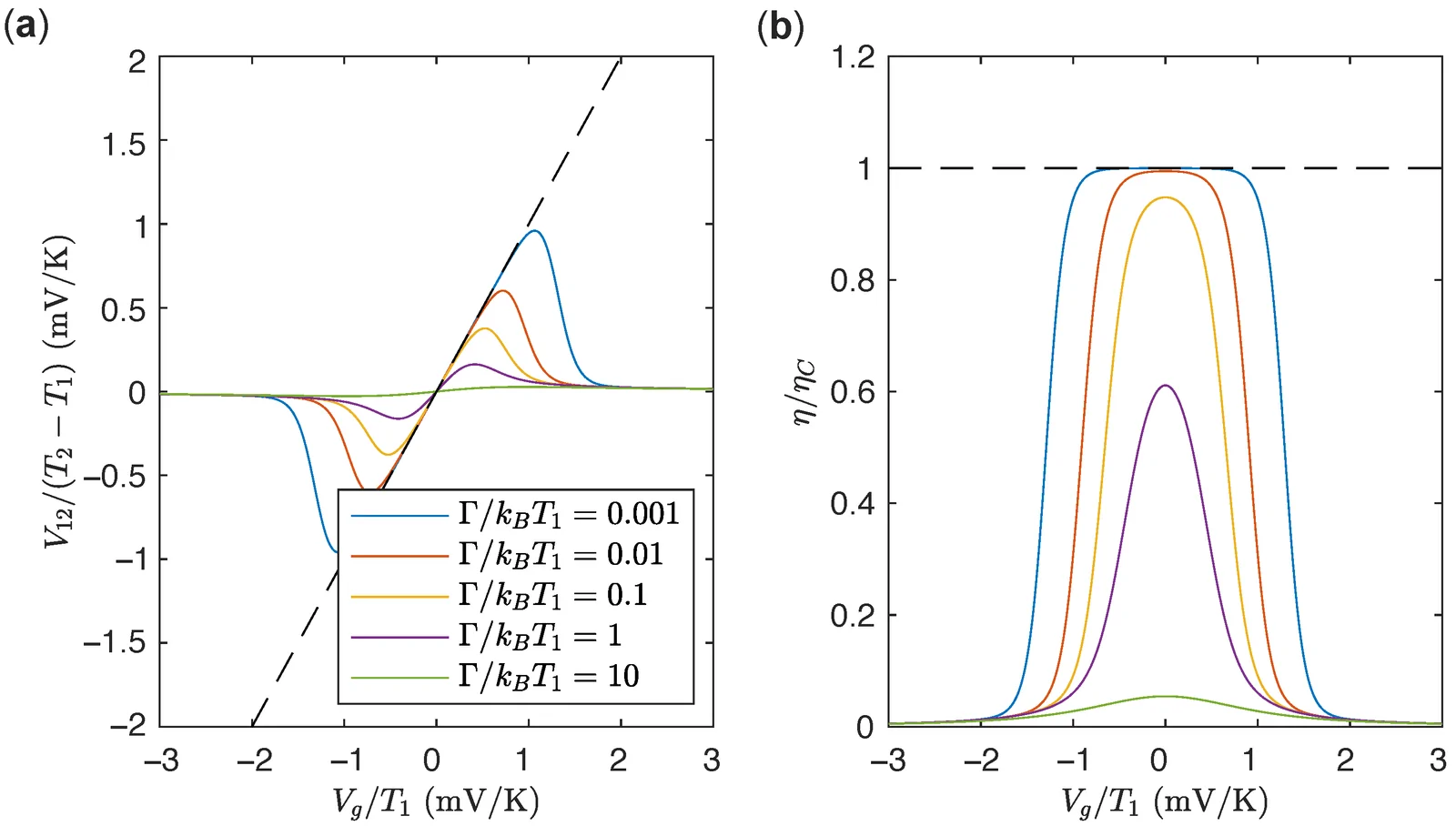
(**a**) Seebeck coefficient $S=-V_{12}/\left(T_{1}-T_{2}\right)$ as a function of the gate voltage $V_{g}$ normalized by the temperature $T_{1}$ for different tunnel coupling strengths. (**b**) The differential efficiency of the quantum dot heat engine relative to the Carnot efficiency for the same tunnel coupling strengths.

Figure 3a shows the Seebeck coefficient, expressed as the open-circuit voltage divided by the temperature difference as a function of the gate voltage relative to the temperature of the hot reservoir, $T_{1}$. For simplicity, we assume the lever arm α=1 and the offset voltage $V_{g0}=0$. The dashed line in Figure 3a corresponds to the Carnot limit where $V_{12}/V_{g}=1-T_{2}/T_{1}$. Figure 3b compares the differential efficiency of the quantum dot heat engine, calculated as $\eta =\partial V_{12}/\partial V_{g}$, to the Carnot efficiency $1-T_{2}/T_{1}$. For weak tunnel coupling relative to the thermal energy, $\Gamma \ll k_{B}T$, the slope of the Seebeck coefficient as a function of gate voltage follows the Carnot limit. As the gate voltage shift the energy level $\epsilon_{0}$ further from the center of the Fermi distribution, the open-circuit voltage tends to zero and the efficiency goes to zero. In the intermediate tunnel coupling regime where $\Gamma ∼k_{B}T$, the efficiency of the quantum dot heat engine does not reach the Carnot limit.

**Figure 4:**
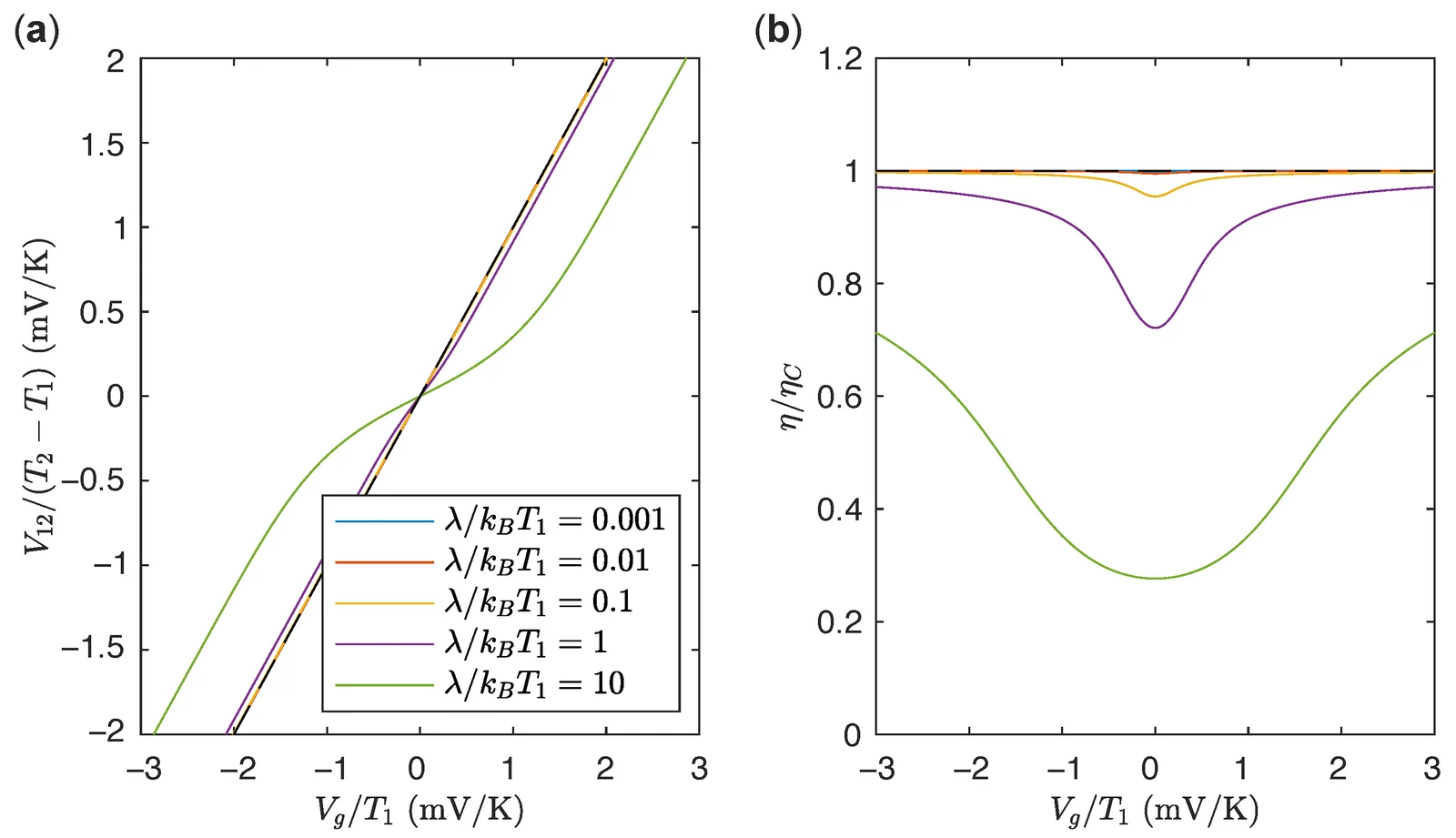
(**a**) Seebeck coefficient $S=-V_{12}/\left(T_{1}-T_{2}\right)$ as a function of the gate voltage $V_{g}$ normalized by the temperature $T_{1}$ for different reorganization energies. (**b**) The differential efficiency of the quantum dot heat engine relative to the Carnot efficiency for the same reorganization energies.

Figure 4a again shows the Seebeck coefficient as a function of gate voltage but for varying electron–vibration coupling strengths given by the reorganization energy λ. In the case of intermediate dissipation, where $\lambda ∼k_{B}T$, the Seebeck coefficient is suppressed around $V_{g}=0$, but at larger detuning, the slope of the Seebeck coefficient as a function of the gate voltage approaches the Carnot limit as shown in Figure 4b. The difference in $V_{12}/V_{g}$ and $1-T_{2}/T_{1}$ in the case of $\lambda ∼k_{B}T$ can be understood as the energy lost to dissipation.

## 3. Discussion

Unsurprisingly, we find that both dissipation and finite tunnel coupling cause the performance of the quantum dot heat engine to diverge from that of a Carnot heat engine. This leaves the question: what is the smallest temperature difference that is detectable using a quantum dot heat engine? Ultimate thermometric precision is often discussed in terms of Fisher information and, in the quantum regime, the quantum Fisher information [36,37]. Since the present treatment is based on classical sequential tunneling and open-circuit transport measurements, we restrict ourselves to the experimentally relevant noise-equivalent temperature estimate. The signal to noise ratio of a quantum dot heat engine will be limited by the current noise across the tunnel junctions, which is on the order of $S_{II}\approx e^{2}\Gamma /\hbar$ [38], and the change in current with temperature $\partial I/\partial T\approx e\Gamma /\hbar T_{1}$, so that the temperature sensitivity is approximately $\sqrt{S_{TT}}=\sqrt{S_{II}}/\left|\partial I/\partial T\right|\approx T_{1}/\sqrt{\Gamma /\hbar }$ [39]. This expression corresponds to the standard noise-equivalent temperature and is equivalent to the Cramér–Rao bound for a Gaussian measurement record. As shown in Figure 3, the tunnel coupling should be less than the thermal energy for the quantum dot heat engine to work as a primary thermometer. Therefore, the smallest detectable temperature difference is approximately $\delta T≳\sqrt{T_{1}\hbar /k_{B}}$, which corresponds to 3 μK/$\sqrt{\mathrm{Hz}}$ at $T_{1}=1$ K. This estimate only includes intrinsic shot-noise fluctuations. In an experimental implementation additional noise sources, such as amplifier noise, charge-noise fluctuations, and slow temperature drifts, will further degrade the achievable sensitivity. The value of 3 μK/$\sqrt{\mathrm{Hz}}$ should therefore be regarded as an optimistic lower bound on the measurable temperature difference.

## 4. Conclusions

We have shown that, in the limit of vanishing dissipation and lifetime broadening, a quantum dot heat engine approaches reversible Carnot operation. Under these conditions, and following independent determination of the relevant device parameters, it provides an operational route towards primary thermometry and the construction of a self-consistent thermodynamic temperature scale. The sensitivity of a quantum dot thermometer is ultimately limited by the statistics of random independent tunneling events.

## Data Availability

The original contributions presented in this study are included in the article. Further inquiries can be directed to the corresponding author.
